# Rehabilitative Exercise Reduced the Impact of Peripheral Artery Disease on Vascular Outcomes in Elderly Patients with Claudication: A Three-Year Single Center Retrospective Study

**DOI:** 10.3390/jcm8020210

**Published:** 2019-02-07

**Authors:** Fabio Manfredini, Nicola Lamberti, Franco Guerzoni, Nicola Napoli, Vincenzo Gasbarro, Paolo Zamboni, Francesco Mascoli, Roberto Manfredini, Nino Basaglia, María Aurora Rodríguez-Borrego, Pablo Jesús López-Soto

**Affiliations:** 1 Department of Biomedical and Surgical Specialties Sciences, University of Ferrara, 44121 Ferrara, Italy; nicola.lamberti@unife.it; 2 Unit of Physical and Rehabilitation Medicine, University Hospital of Ferrara, 44121 Ferrara, Italy; nino.basaglia@unife.it; 3 Department of Nursing, Maimonides Biomedical Research Institute of Cordoba (IMIBIC), University of Cordoba, Reina Sofía University Hospital, 14004 Cordoba, Spain; mfr@unife.it (R.M.); en1robom@uco.es (M.A.R.-B.); pablo.lopez@imibic.org (P.J.L.-S.); 4 Department of Medical Statistics, University Hospital of Ferrara, 44124 Ferrara, Italy; f.guerzoni@ospfe.it (F.G.); n.napoli@ospfe.it (N.N.); 5 Unit of Vascular and Endovascular Surgery, University Hospital of Ferrara, 44124 Ferrara, Italy; vincenzo.gasbarro@unife.it (V.G.); francesco.mascoli@unife.it (F.M.); 6 Unit of Translational Surgery, University Hospital of Ferrara, 44124 Ferrara, Italy; zambo@unife.it; 7 Department of Medical Sciences, University of Ferrara, 44121 Ferrara, Italy

**Keywords:** mortality, exercise, peripheral artery disease, rehabilitation, vascular surgical procedures

## Abstract

The study retrospectively evaluated the association between rehabilitative outcomes and risk of peripheral revascularizations in elderly peripheral artery disease (PAD) patients with claudication. Eight-hundred thirty-five patients were enrolled. Ankle-brachial index (ABI) and maximal walking speed (S_max_) were measured at baseline and at discharge from a structured home-based rehabilitation program. For the analysis, patients were divided according to a baseline ABI value (severe: ≤0.5; moderate: ≥0.5) and according to hemodynamic or functional rehabilitative response (responder: ABI ≥ 0.10 and/or S_max_ > 0.5 km/h). Three-year outcomes were collected from the regional registry. According to the inclusion criteria (age 60–80, ABI < 0.80; program completion) 457 patients, 146 severe and 311 moderate, were studied. The whole population showed significant functional and hemodynamic improvements at discharge, with 56 revascularizations and 69 deaths at follow-up. Compared to the moderate group, the severe group showed a higher rate of revascularizations (17% vs. 10%, *p* < 0.001) and deaths (29% and 8%, respectively; *p* < 0.001). However, patients with severe PAD who were ABI responders after rehabilitation showed less revascularizations than non-responders (13% vs. 21%; hazard ratio (HR): 0.52) and were not different from patients with moderate disease (9%). Superimposable rates were observed for S_max_ responders (13% vs. 21%; HR: 0.55; moderate 10%). In conclusion, elderly patients with severe PAD empowered by better rehabilitation outcomes showed lower rates of peripheral revascularizations and deaths that were comparable to patients with moderate PAD.

## 1. Introduction

Peripheral artery disease (PAD), which is particularly prevalent in the elderly, affects more than 200 million people worldwide, and the incidence is continuously rising [1]. PAD patients suffer reduced mobility due to classical and atypical symptoms and they are at high risk of cardiovascular events and deaths, most commonly due to cardiovascular causes [2]. Progressively lower ankle-brachial index (ABI) values and higher functional disability in these patients are associated with worse vascular and cardiovascular outcomes [2,3,4,5,6,7]. An invasive approach in the elderly in the presence of severe disease and claudication may not guarantee functional and/or quality of life improvements and may increase the risk of complications, especially in the presence of comorbidities [8,9,10,11]. On the other hand, a conservative approach may cause deconditioning due to physical inactivity with associated reduced quality of life and higher risk of hospitalizations and mortality [12,13,14,15]. Exercise therapy is recommended at intermediate stages of PAD [1], and it may promote a non-invasive improvement in walking ability and quality of life in elderly patients with claudication [1]. Unfortunately, recommended supervised rehabilitation programs may not be available, despite being effective [16]. When available, these programs may be associated with insufficient adherence because of problems with costs and transportation, or may exclude patients with more severe disease and disability [17,18,19,20].

More than 10 years ago, a structured, home-based, pain-free program that enrolled PAD patients with severe disease showed mobility and hemodynamic improvements [11,21,22,23]. The program was adapted to frail subjects and improved the mobility of stroke survivors [24,25] and end-stage renal disease patients with better long-term outcomes [26,27,28,29]. Unfortunately, the long-term outcomes of rehabilitation in PAD are poorly described [30].

We hypothesize that the functional and hemodynamic adaptations evoked by a structured aerobic intervention would favor long-term vascular outcomes, even in severe PAD. The present single-center retrospective study tested this hypothesis in a cohort of elderly patients and determined whether a rehabilitation program altered the rate of PAD-related revascularization and mortality three years post-discharge.

## 2. Materials and Methods

### 2.1. Study Population

This single-center study retrospectively analyzed a prospectively collected database of PAD patients who were referred to the vascular rehabilitation program at the University Hospital of Ferrara between 2005 and 2013. The local ethics committee approved the study and written informed consent was not obtained from patients who were no longer attending the program.

A total of 835 consecutive patients with PAD at Fontaine’s stage II who were previously diagnosed at the Department of Vascular Surgery were enrolled in the study.

### 2.2. Exercise Program

All patients received the “test in–train out” home-based exercise program [21,22]. The structured exercise was prescribed at the hospital during circa-monthly visits, but it was executed at home. The program encompassed two daily 10-minute sessions of intermittent walking (one-minute walk followed by one-minute seated rest) for six days each week at a prescribed speed. The training speed was converted into a walking cadence (steps/minute) and paced at home by using a metronome. It was slower than the individual’s walking speed at the beginning and increased weekly. A record of the training sessions was requested and collected at each visit. The patients were discharged from the program when a satisfactory and/or stable improvement in pain-free walking distance was attained. More details on the exercise program are reported elsewhere [21,22].

### 2.3. Outcomes

PAD-related lower limb revascularization was the primary outcome and all-cause mortality was another outcome of interest. Outcomes were considered after the date of participants’ discharge from the program. The clinical data for a 3-year follow-up period after the discharge date were gathered from the Emilia-Romagna health service registry.

### 2.4. Study Variables

The ABI was measured at the beginning of, during, and at discharge from the program according to the established standards [1]. An incremental treadmill test based on level walking [31] was performed to determine the speed at onset of symptoms and the maximal speed (S_max_) attainable for each patient.

The patients who did not complete the rehabilitation program, had an ABI >0.8 at baseline or was non-measurable, were aged <60 or >80 years, or had missing/incomplete information regarding long-term outcomes, were excluded.

To perform all the analyses, the final PAD population was divided into two groups according to disease severity at entry: severe (Sev), for ABI values ≤0.5 in the more impaired limb and moderate (Mod) for ABI values >0.5.

The improvement obtained at the end of the program compared to the baseline was categorized according to previous prospective studies for further analyses [11,21,23]. The following favorable outcomes were considered: for hemodynamics, an ABI value increase ≥0.10 for the more impaired limb; and for functional capacity, S_max_ variations ≥0.5 km/h.

### 2.5. Statistical Analysis

Differences in baseline characteristics were assessed according to PAD severity. Differences were assessed using chi-squared tests, Student’s *t*-test, or the Mann-Whitney U-test as appropriate. Logistic regression with a stepwise selection method was used to identify the factors related to a non-response to rehabilitation.

Kaplan-Meier estimates of the distribution of times from discharge to the clinical events and a log-rank test for trend were used to compare the curves of the four patient subgroups (Sev and Mod, with/without hemodynamic or functional improvement). Data for peripheral revascularization were censored at the time of death.

Univariate and multivariate Cox proportional hazards regression analyses were used to analyze the effect of several predictor variables on the primary outcome in the entire population and in each of the two groups. Because of the limited number of events, multivariate hazard ratios (HRs) were calculated using a forward approach, with an entry limit of *p* < 0.05.

A *p* value of <0.05 was considered statistically significant. All statistical analyses were performed using MedCalc Statistical Software version 18.10 (MedCalc Software bvba, Ostend, Belgium). Research data are available at: http://dx.doi.org/10.17632/4536z7c3nk.1.

## 3. Results

**Figure 1:**
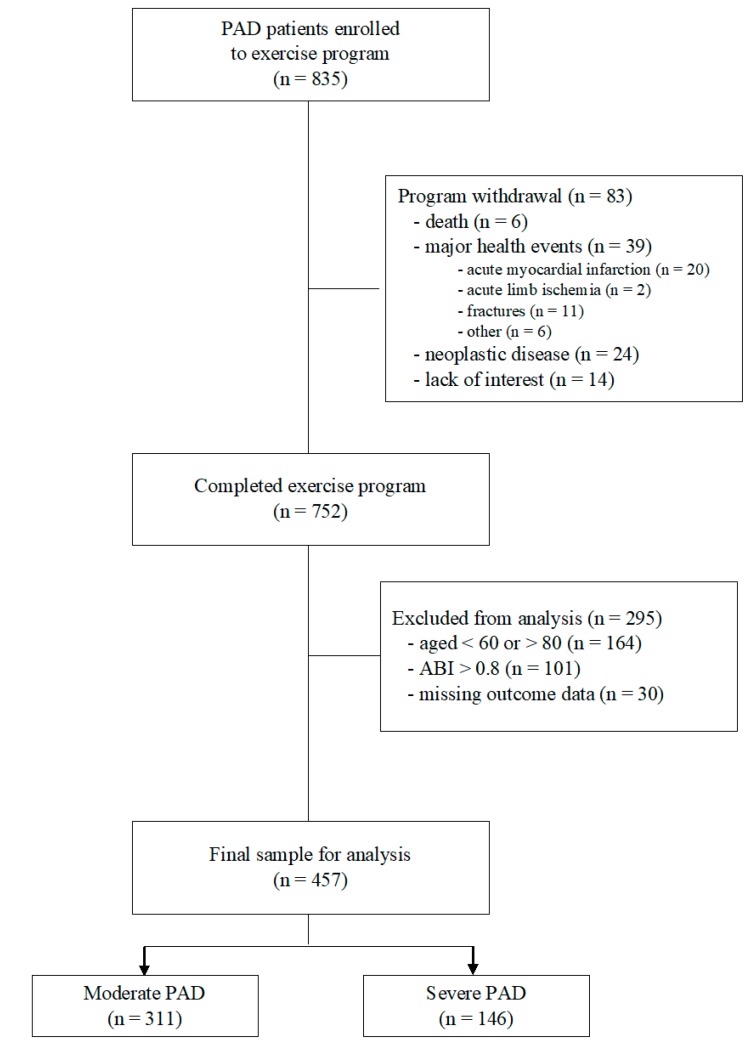
Flow diagrams of participants. Abbreviations: PAD, peripheral artery disease; ABI, ankle-brachial index.

From January 2005 to January 2013, 835 patients with PAD were enrolled in the rehabilitation program. A flow diagram of the participants and the reasons for exclusion are reported in Figure 1.

The final sample for this study included 457 elderly patients; 146 patients were Sev and 311 patients were Mod. The baseline demographics and clinical characteristics of the two subgroups, which differed by more prevalent bilateral disease in Sev, both limbs’ ABI and functional capacity, are shown in Table 1.

### 3.1. Exercise Program

All patients completed the exercise program, which lasted 394 ± 177 days, but the number of days was significantly greater in the Sev group than the Mod group (433 ± 188 vs. 376 ± 169 days, respectively; *p* = 0.001). Adherence to the program at a controlled speed was high for both groups, with both executing 87% of walking sessions prescribed (*p* = 0.77).

Significant improvements in the ABI of both limbs and S_max_ were observed at the end of the program in the entire population and both groups. Significant between-group differences were found for both ABI values in favor of the Sev group (<0.001). (Table 2, Supplementary Figure S1).

For long-term outcome analyses, 71 Sev patients (49%) improved to ABI ≥ 0.10 (Sev_ABI+_) and 75 (51%) achieved enhanced S_max_ ≥0.5 km/h (Sev_Smax+_). Eighty-eight (28%) of the Mod patients had improved ABI (Mod_ABI+_) and 164 (53%) achieved enhanced S_max_ (Mod_Smax+_).

The regression models for the entire population and both groups, including the independent variables related to subjects, risk factors, comorbidities, and PAD characteristics (Table 1), did not identify any factors related to a non-response to rehabilitation when ABI and S_max_ improvements were considered.

### 3.2. Primary Outcome: PAD-Related Revascularizations

Fifty-six (12%) patients in the whole population had undergone peripheral revascularization at the 3-year follow-up; 25 from the Sev group (17%) and 31 from the Mod group (10%) (log-rank *p* = 0.006).

**Figure 2:**
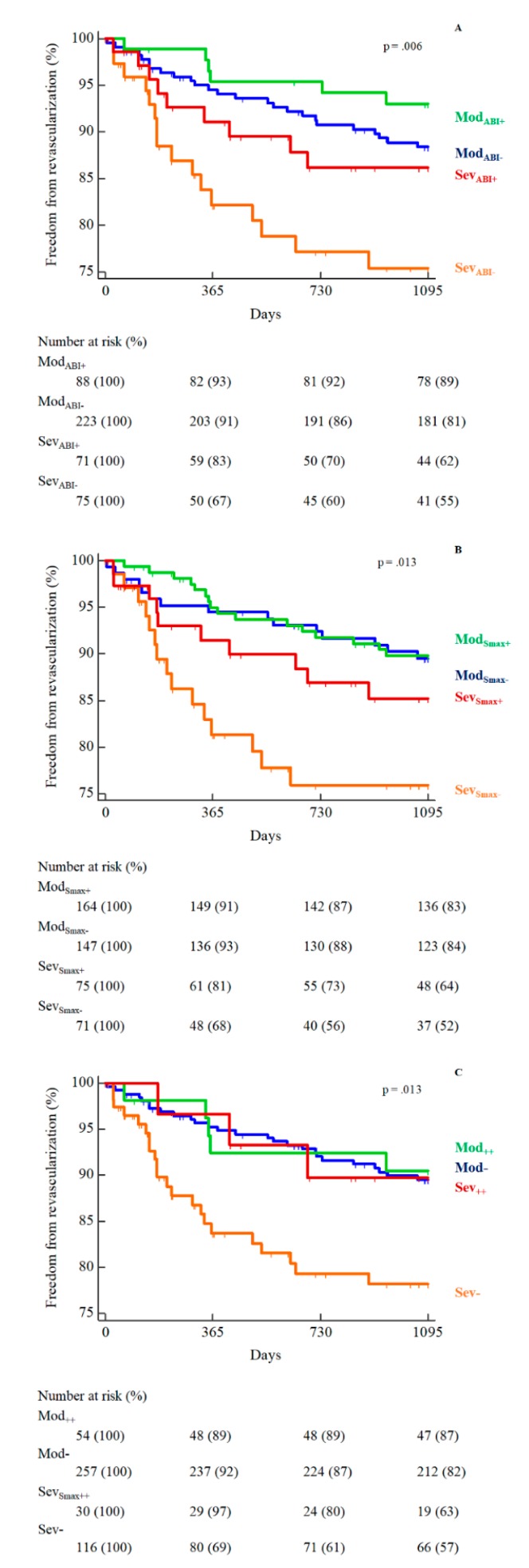
Kaplan-Meier curves of revascularizations in the four patients’ subgroups according to disease severity and ABI (**A**), or maximal speed improvements (**B**), or both (**C**).

Considering the rehabilitative outcomes, analyses of the ABI change showed a 3-year revascularization rate of 21% in Sev_ABI−_, 13% in Sev_ABI+_, 11% in Mod_ABI−_ and 7% in Mod_ABI+_ (Figure 2A). Similar rates were observed for functional capacity improvements, particularly 21% in Sev_Smax−_, 13% in Sev_Smax+_, and 10% in both Mod subgroups (Figure 2B).

For both analyses within the Sev subgroups, the hemodynamic or functional improvement resulted in a protective, although not statistically significant, HR of 0.52 (0.20–1.40) and of 0.55 (0.20–1.50), respectively, which was enhanced in the case of concomitant improvements of the two outcomes (HR 0.43; 0.13–1.36) (Figure 2C).

### 3.3. Secondary Outcome

There were 69 deaths (15%) during the follow-up period; 43 in the Sev and 26 in the Mod group (29% vs. 8%, respectively, log-rank *p* < 0.001).

**Figure 3:**
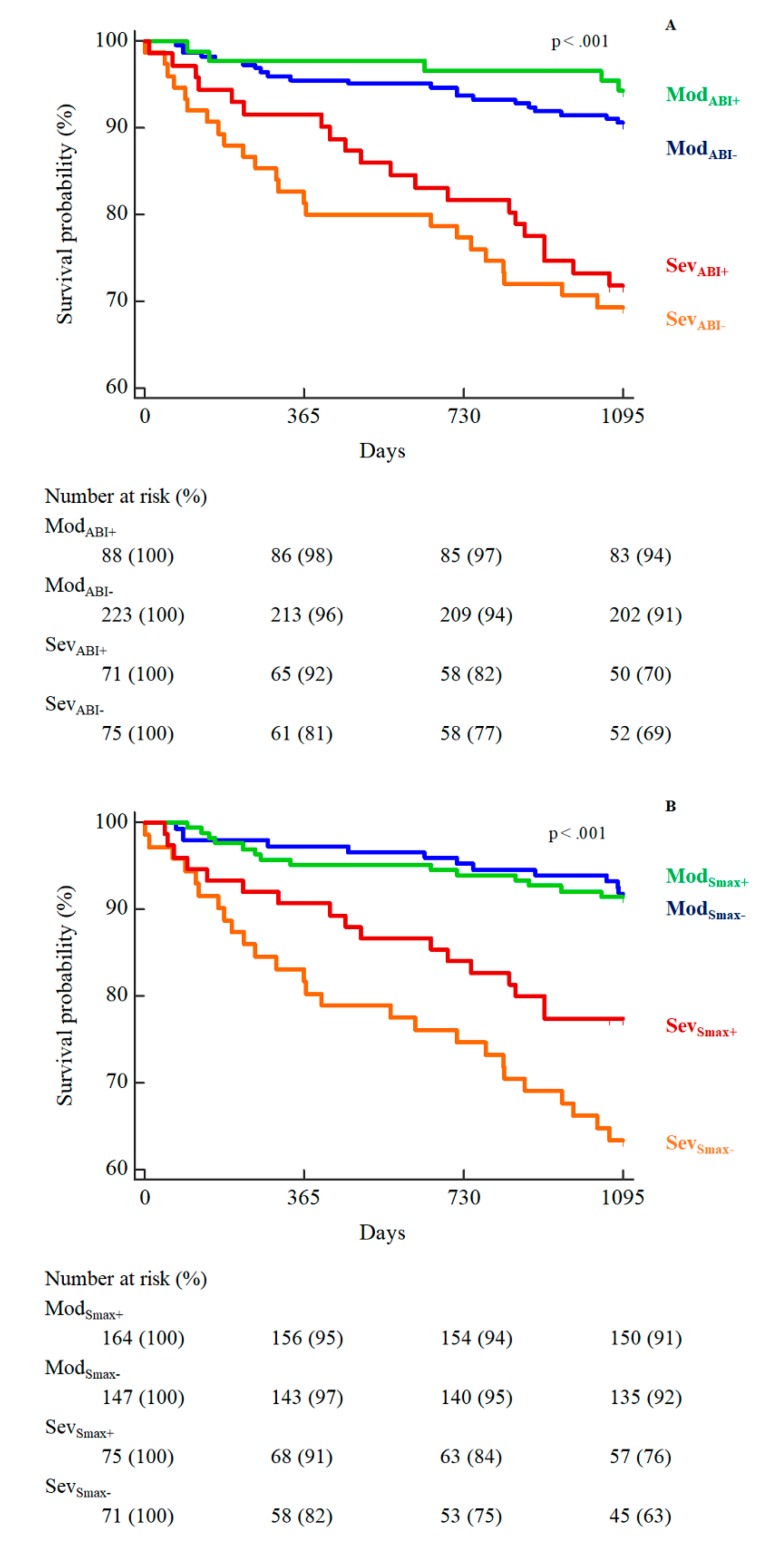
Kaplan-Meier curves of survival in the four patients’ subgroups according to disease severity and ABI (**A**), or maximal speed improvements (**B**).

Higher mortality rates were observed for Sev_ABI−_ (31%) compared to the other subgroups, including Sev_ABI+_ (28%), Mod_ABI−_ (9%), and Mod_ABI+_ (6%) (Figure 3A).

Similarly, the subgroup Sev_Smax-_ showed a higher mortality at 37% compared to Sev_Smax+_ (23%), Mod_Smax−_ (8%), and Mod_Smax+_ (9%) (Figure 3B). No statistically significant differences were observed between the Sev subgroups.

### 3.4. Predictors of Revascularization

**Figure 4:**
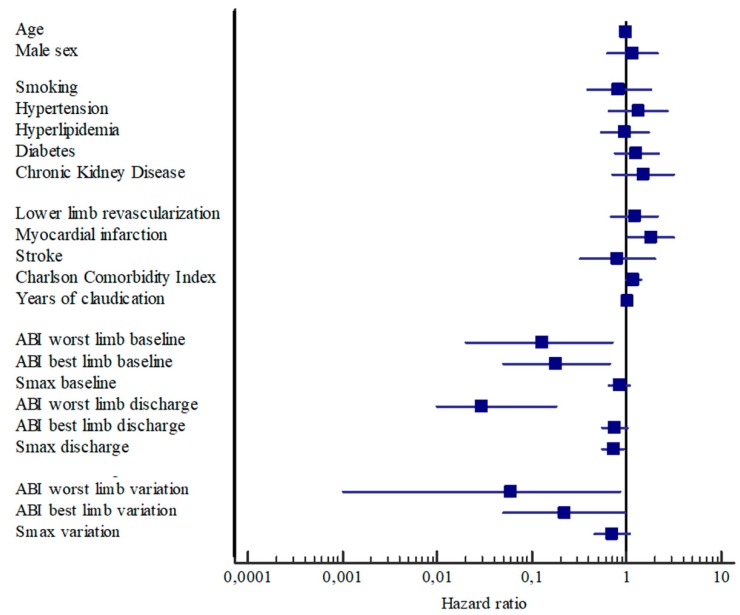
Forest plot showing association between PAD-related revascularizations and study variables in the whole population.

History of myocardial infarction (HR: 1.90; 1.07–3.36) and the ABI value of the more impaired limb at discharge (HR: 0.03; 0.004–0.16) were the only predictors of peripheral revascularizations in the entire population according to multivariate Cox regression. The univariate analyses highlighted the impact of baseline ABI values and their changes following rehabilitation (Table 3, Figure 4).

Several independent predictors were identified in the Sev group, but the ABI improvement in the more impaired limb was the most protective factor against peripheral revascularization (HR: 0.003; 0.0001–0.09), which reduced the risk by greater than 300%.

Only chronic kidney disease and the ABI of the more impaired limb at discharge were included in the analysis in the Mod group (Table 3, Supplementary Figure S2).

## 4. Discussion

This retrospective study shows for the first time that hemodynamic and functional changes following a rehabilitation program in a population of elderly PAD patients are associated with a significant reduction in the risk of PAD-related revascularization at a three-year follow-up. Notably, the severe PAD patients who attained some degree of hemodynamic and functional changes showed a lower risk of limb revascularizations that was comparable to patients with moderate disease. As a starting point, the present study confirmed the effectiveness of a structured, home-based program [11,21,22] on exercise capacity in the entire population and both PAD subgroups. Patients with severe disease and significantly lower functional values at baseline achieved the same improvements as patients with moderate disease. A significant ABI increase was also observed in the entire population and both PAD subgroups, which has been observed in previous studies [11,21,22,23,32,33] and poorly reported following supervised programs [16]. Notably, hemodynamic changes were more evident in the severe PAD patients. However, the most significant aspect of the study reveals an important, yet poorly reported, effect of rehabilitation on long-term outcomes and particularly the vascular interventions in PAD.

The clinical outcomes of the entire PAD population under study after three years were comparable or even better than previous reports in the literature [34,35,36]. A rate of 12% of vascular intervention (2.6% for acute limb ischemia) was observed, with 0.8% amputations and 15% deaths. As expected and previously reported [4,6,7], worse vascular and clinical outcomes at three years were observed in PAD patients with severe disease, with a 2−3-fold higher rate of events compared to patients with moderate disease (17% vs. 10% for interventions and 8% vs. 29% of deaths, respectively).

Previous studies reported negative outcomes (deaths, functional decline, re-interventions) associated with low exercise capacity [15,37], negative changes of ABI over time [5,38], and rehabilitation poorly attended [30] or not combined to revascularization [10].

Notably, the present study showed that it was not the functional and hemodynamic values at baseline, but the variations in these factors following rehabilitation that were primarily associated with the fate of elderly patients. Namely, the improvements observed at discharge from the program reduced the relative risk of lower limb revascularizations at a three-year follow-up, especially in the population with severe disease. The ABI improvement of the more impaired limb in this group of patients represented the most protective factor against peripheral revascularization, with a reduction in risk of over 300%. In particular, an ABI improvement ≥0.10 was a relevant outcome that was associated with a lower risk of future interventions. This hemodynamic change, associated with personalized aerobic training, may have favored a significant gain in mobility in a subgroup of severe patients. Those who attained hemodynamic and functional improvements showed vascular outcomes that were almost comparable to the moderate group and a lower mortality rate. The data collected highlight the role of rehabilitation, considering that interventional procedures may be not effective or satisfactory for mobility [9,10] in patients with intermittent claudication.

The present study demonstrated the sustainability of the program, which bypasses most barriers of the supervised programs [17,20]. The program is without gender differences [39] or limitations from pre-existing osteoarticular pathologies [40] and enables the enrolment of frail patients with restricted mobility. This exercise is based on over-ground personalized walking sessions inside the home and it is safe and painless [19]. The duration of the program, which was deliberately made longer in the severe group, allows for a protective monitoring of lifestyle and therapy adherence [41]. The program calls for few hospital visits, which maintains a low cost for the National Health Service [11,22].

This study has several limitations. First, its retrospective design may have influenced data recording. However, the same team prospectively collected the data from patients enrolled consecutively, and the data were secondarily elaborated and analyzed by personnel who were not involved in the study. A control group not exposed to rehabilitation is absent, but the objective was to explore the long-term response to the measured hemodynamic and functional effects of rehabilitation. The cut-off values for the analyses of the rehabilitative outcomes were arbitrary but in accordance with previous studies [11,21,23]. Patients with non-measurable ABI were excluded because it was not possible to classify them according to hemodynamic severity. Patients aged over 80 years were also excluded on the basis of the life expectancy in our province (average of 82.2 years).

Finally, a treadmill test was used instead of an over-ground walking test to favor standardization.

## 5. Conclusions

A structured home-based rehabilitation program evoking functional and hemodynamic improvements reduced the long-term risk of vascular outcomes and deaths in elderly patients with claudication and in the presence of severe PAD.

Patient-centered programs may represent an option in the decision-making for elderly patients with low mobility and severe disease, but a prospective study is warranted to confirm the data presented in this study.

## Figures and Tables

**Table 1:** Baseline characteristics of patients included in the analysis.

	Moderate (*n* = 311)	Severe (*n* = 146)	*p*
Male sex	223 (72)	110 (75)	0.41
Age, years	71 ± 6	72 ± 5	0.07
Sedentary occupation	209 (67)	89 (61)	0.19
Risk factors; *n* (%)			
Smoking	277 (89)	134 (92)	0.37
Hypertension	247 (79)	121 (83)	0.38
Hyperlipidemia	218 (70)	103 (71)	0.92
Diabetes mellitus	119 (38)	54 (37)	0.79
Chronic Kidney Disease	30 (10)	20 (14)	0.20
Familiarity for CVD	72 (23)	35 (24)	0.85
Comorbidities; *n* (%)			
Chronic Heart Disease	123 (40)	63 (43)	0.47
Stroke	35 (11)	16 (11)	0.93
Osteoarticular disease	78 (25)	35 (24)	0.80
Pulmonary disease	18 (6)	15 (10)	0.08
Neoplastic disease	25 (8)	14 (10)	0.58
Charlson Comorbidity Index	2.6 ± 1.4	2.7 ± 1.5	0.51
Age-adjusted Charlson Index	6.2 ± 1.5	6.4 ± 1.6	0.27
Peripheral artery disease			
Grade I—Category 1	168 (54)	31 (21)	<0.001
Grade I—Category 2	118 (38)	55 (38)	<0.001
Grade I—Category 3	25 (8)	60 (41)	<0.001
Self-reported claudication distance (m)	209 ± 187	114 ± 121	<0.001
Lower limbs revascularization	85 (27)	49 (34)	0.17
Disease duration, years	6 ± 6	7 ± 6	0.09
Bilateral disease	206 (66)	119 (82)	<0.001
ABI more impaired limb	0.64 ± 0.08	0.39 ± 0.10	<0.001
ABI less impaired limb	0.86 ± 0.16	0.66 ± 0.22	<0.001
PTS (km/h)	2.9 ± 1.1	2.5 ± 0.9	<0.001
S_max_ (km/h)	3.4 ± 1.1	3.0 ± 1.0	<0.001

Abbreviations: CVD, cardiovascular disease; ABI, ankle-brachial index; PTS, speed at symptoms; S_max_, maximal speed. Legend: Disease severity is reported according to Rutherford classification.

**Table 2:** Within- and between-group differences in rehabilitation outcomes.

	Moderate (*n* = 311)				Severe (*n* = 146)					
	Baseline	End	∆	*p* Within-Group	Baseline	End	∆	*p* Within-Group	Between-Group ∆ in Changes	*p* Between-Group
**ABI worst leg**	0.64 (0.63–0.65)	0.69 (0.67–0.70)	0.04 (0.03–0.05)	<0.001	0.39 (0.38–0.41)	0.50 (0.48–0.52)	0.11 (0.09–0.12)	<0.001	0.06 (0.04–0.08)	<0.001
**ABI best leg**	0.86 (0.84–0.88)	0.89 (0.88–0.90)	0.03 (0.02–0.04)	<0.001	0.66 (0.63–0.70)	0.72 (0.68–0.76)	0.06 (0.03–0.09)	<0.001	0.03 (0.01–0.05)	<0.001
**PTS (km/h)**	2.9 (2.8–3.0)	3.7 (3.5–3.8)	0.8 (0.7–0.9)	<0.001	2.4 (2.3–2.6)	3.1 (3.0–3.3)	0.7 (0.6–0.8)	<0.001	0.1 (−0.1–0.2)	0.23
**S** **_max_** **(km/h)**	3.4 (3.3–3.6)	4.0 (3.8–4.1)	0.5 (0.4–0.6)	<0.001	3.0 (2.9–3.2)	3.4 (3.3–3.6)	0.4 (0.3–0.5)	<0.001	0.1 (−0.2–0.2)	0.10

Abbreviations: ABI—ankle-brachial index; PTS—speed at symptoms; S_max_—maximal speed. Legend: Data are expressed as mean and 95% Confidence Interval.

**Table 3:** Results of Cox proportional hazards regression analyzing the capability of the study variables for the prediction of 3-year revascularization in the whole population and in the two patient groups.

	Whole Population (*n* = 457)		Moderate (*n* = 311)		Severe (*n* = 146)	
	*Univariate*	*Multivariate*	*Univariate*	*Multivariate*	*Univariate*	*Multivariate*
	*HR (95% CI)*	*HR (95% CI)*	*HR (95% CI)*	*HR (95% CI)*	*HR (95% CI)*	*HR (95% CI)*
Age	0.99 (0.94–1.04)		0.97 (0.91–1.03)		1.01 (0.93–1.08)	
Male sex	1.15 (0.63–2.12)		1.76 (0.72–4.30)		0.64 (0.28–1.50)	
Smoking	0.83 (0.38–1.83)		0.86 (0.30–2.46)		0.72 (0.21–2.39)	
Hypertension	1.31 (0.64–2.69)		1.36 (0.52–3.56)		1.16 (0.40–3.39)	
Hyperlipidemia	0.96 (0.54–1.69)		1.75 (0.71–4.26)		0.52 (0.24–1.15)	0.24 (0.10–0.60)
Diabetes mellitus	1.27 (0.74–2.15)		1.57 (0.78–3.19)		0.95 (0.42–2.14)	
Chronic Kidney Disease	1.50 (0.71–3.18)		2.50 (1.03–6.12)	2.99 (1.20–7.45)	0.60 (0.14–2.54)	
Lower limbs revascularization	1.21 (0.69–2.12)		1.81 (0.88–3.72)		0.62 (0.25–1.57)	
Myocardial infarction	1.80 (1.02–3.19)	1.90 (1.07–3.36)	1.75 (0.82–3.73)		2.20 (0.92–5.27)	3.63 (1.44–9.14)
Stroke	0.79 (0.31–1.99)		0.89 (0.27–2.93)		0.66 (0.16–2.79)	
Charlson Comorbidity Index	1.19 (1.00–1.42)		1.22 (0.97–1.54)		1.12 (0.88–1.45)	
Disease duration	1.02 (0.98–1.06)		0.99 (0.93–1.06)		1.04 (0.99–1.10)	
ABI worst limb baseline	0.13 (0.02–0.70)		0.21 (0.002–20.62)		1.52 (0.02–82.29)	
ABI best limb baseline	0.18 (0.05–0.66)		3.46 (0.42–28.60)		0.06 (0.01–0.44)	0.02 (0.001–0.22)
S_max_ baseline	0.84 (0.65–1.07)		0.92 (0.67–1.27)		0.81 (0.54–1.21)	
ABI worst limb discharge	0.03 (0.006–0.18)	0.03 (0.004–0.16)	0.05 (0.002–0.98)	0.02 (0.001–0.42)	0.04 (0.002–0.93)	
ABI best limb discharge	0.76 (0.55–1.02)		0.64 (0.33–1.16)		0.82 (0.24–2.92)	
S_max_ discharge	0.72 (0.56–0.93)		0.85 (0.59–1.21)		0.69 (0.47–1.01)	0.57 (0.37–0.89)
Δ ABI worst limb	0.06 (0.004–0.84)		0.03 (0.001–1.29)		0.01 (0.0002–0.45)	0.003 (0.0001–0.09)
Δ ABI best limb	0.22 (0.05–0.97)		0.20 (0.04–1.47)		0.23 (0.03–1.66)	
Δ S_max_	0.70 (0.45–1.08)		0.85 (0.49–1.49)		0.57 (0.29–1.14)	

## Supplementary Materials

The following are available online at http://www.mdpi.com/2077-0383/8/2/210/s1, Figure S1: More impaired limb ankle-Brachial indexes and maximal speed distribution in the whole population at baseline (blue) and at discharge (orange), Figure S2: Forest plots showing association between PAD-related revascularizations and study variables in the moderate (A) and severe (B) groups.
