# Supplementary material for: Rehabilitative Exercise Reduced the Impact of Peripheral Artery Disease on Vascular Outcomes in Elderly Patients with Claudication: A Three-Year Single Center Retrospective Study
Source: J Clin Med. 2019 Feb 7;8(2):210. doi: 10.3390/jcm8020210 (PMC6406499; doi:10.3390/jcm8020210)

Supplementary Figure S1: More impaired limb Ankle-Brachial Indexes and maximal speed distribution in the whole population at baseline (blue) and at discharge (orange).

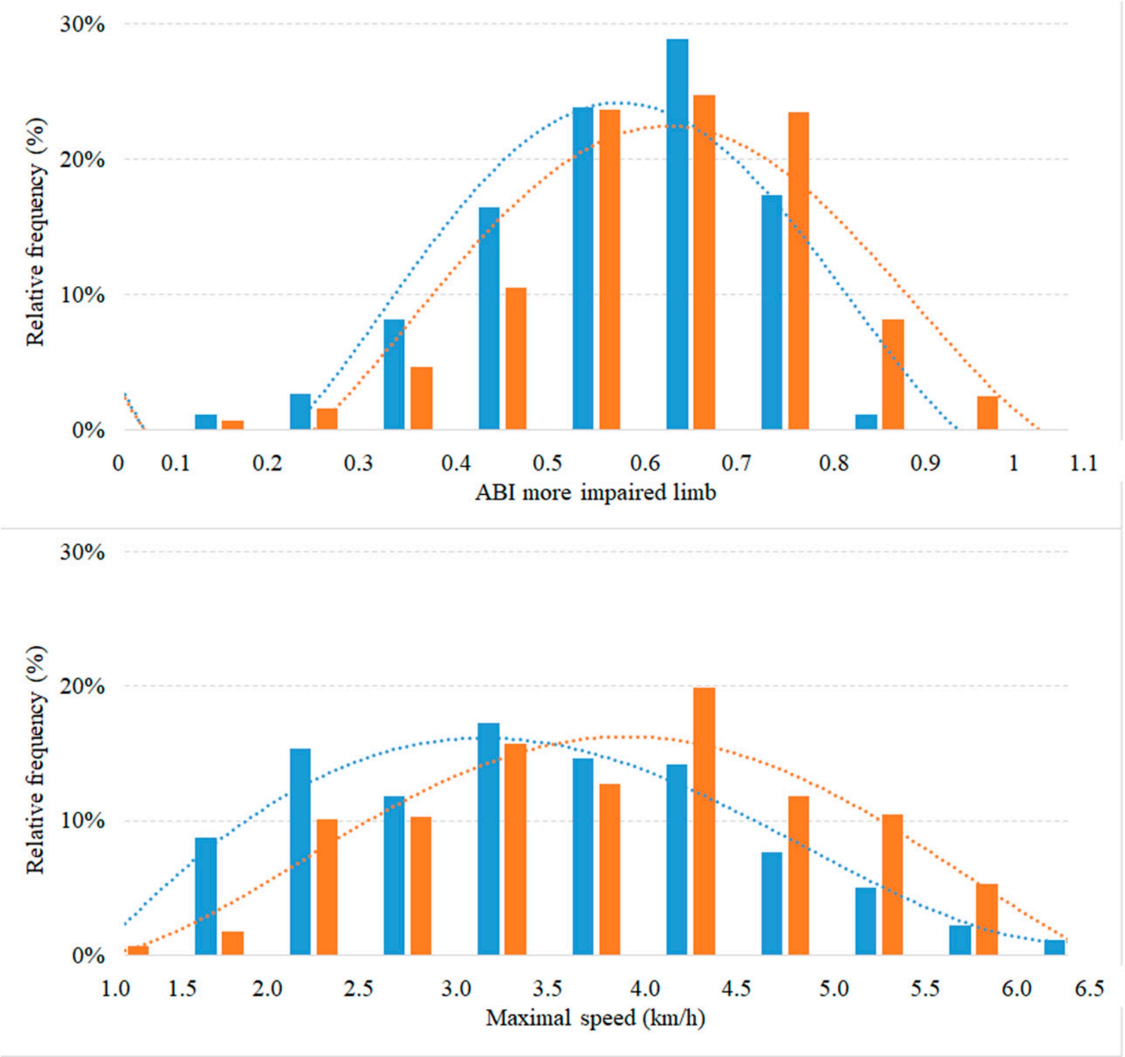

Supplementary Figure S2: Forest plots showing association between PAD-related revascularizations and study variables in the Moderate (A) and Severe (B) groups.

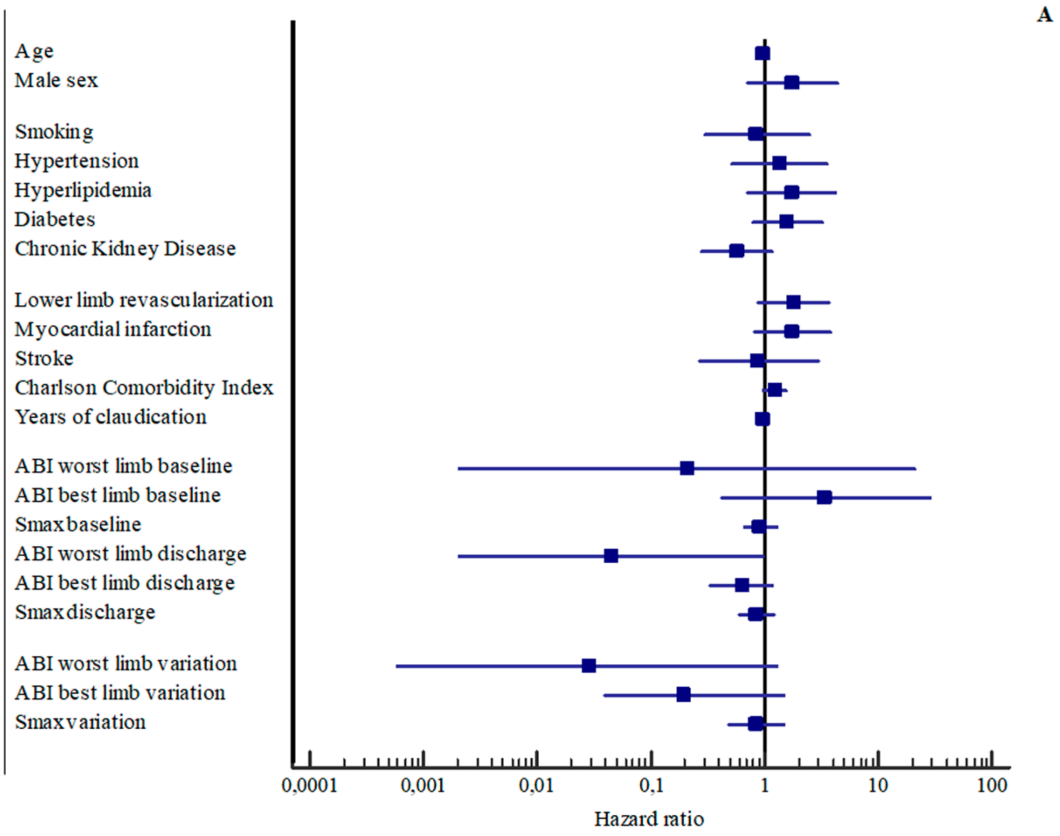

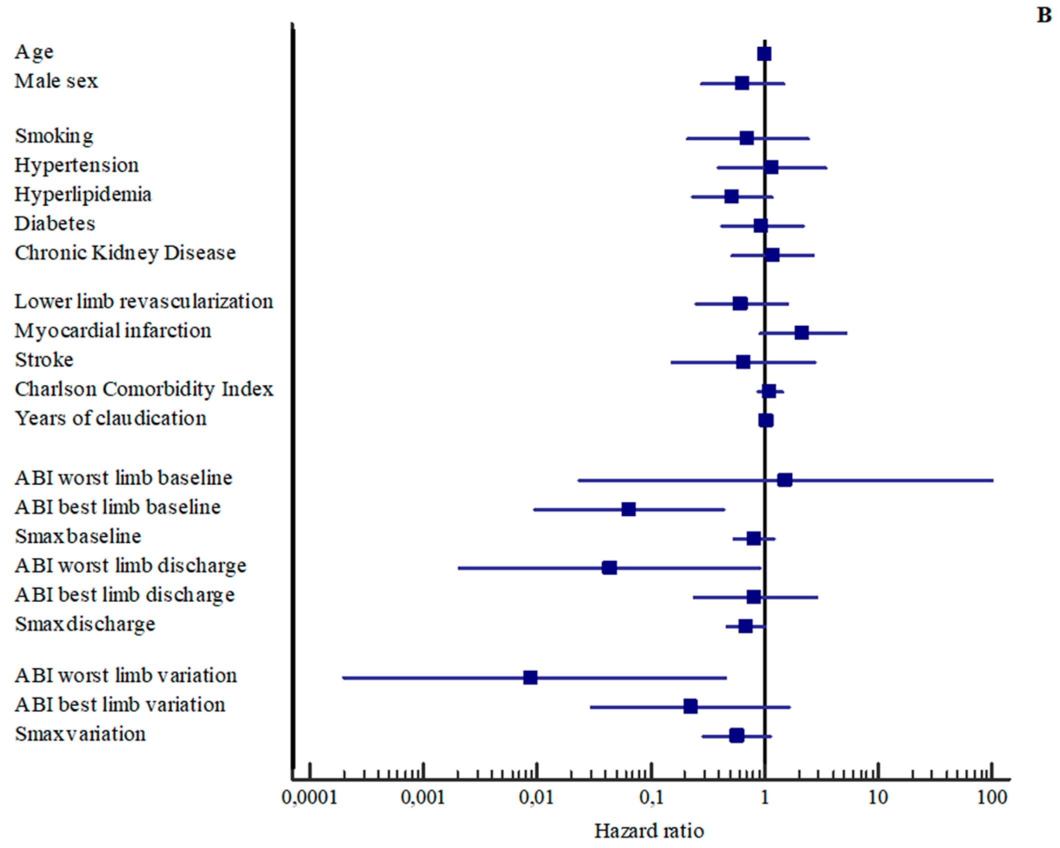

Supplement: Supplementary file 1 [file jcm-08-00210-s001.pdf]
